# Ceramic-Based Composite Membrane with a Porous Network Surface Featuring a Highly Stable Flux for Drinking Water Purification

**DOI:** 10.3390/membranes9010005

**Published:** 2019-01-02

**Authors:** Li Zhu, Kadalipura Puttaswamy Rakesh, Man Xu, Yingchao Dong

**Affiliations:** 1 Engineering Research Center of Environmental Materials and Membrane Technology of Hubei Province, School of Materials Science and Engineering, Wuhan Institute of Technology, Wuhan 430205, China; lzhu@wit.edu.cn (L.Z.); rakesh@whut.edu.cn (K.P.R.); xuman@wit.edu.cn (M.X.); 2 Key Laboratory of Industrial Ecology and Environmental Engineering (Ministry of Education, MOE), School of Environmental Science and Technology, Dalian University of Technology, Dalian 116024, China

**Keywords:** water purification, ceramic membrane, carbon nanotube, bacterial removal, highly stable flux, high rejection

## Abstract

Highly efficient drinking water purification is still an important challenge for membrane techniques where high flux, high rejection, and low fouling are highly emphasized. In the present work, a porous network surface with carbon nanotubes (CNTs) was in situ constructed on hierarchically-structured mullite ceramic membranes. Interestingly, such a composite structure was demonstrated to effectively remove bacteria from drinking water with a highly stable long-term flux. After membrane structure characterizations, separation performance, such as flux and rejection, was assessed by the purification of bacteria-contaminated drinking water. The results confirmed that the mullite-CNT composite membrane claimed a complete removal of two model bacteria (100% rejection of *Escherichia coli* (*E. coli*) and *Staphylococcus aureus* (*S. aureus*)), driven by a trans-membrane pressure of 0.1 MPa, where a surface sieving mechanism was dominant. A highly stable long-term flux for the 24 h filtration process was achieved, which can be attributed to the porous membrane surface with a special randomly-oriented CNTs network structure, featuring very high three-dimensional open porosity, allowing water to rapidly transport. The bacteria were only trapped on the CNTs network surface via surface filtration, without pore plugging, endowing the mullite-CNT membrane with unprecedentedly low fouling propensity to keep high flux with long-term operation time.

## 1. Introduction

Water-borne pathogens such as bacteria, protozoans, helminths, fungi, and viruses are a major health threat and; therefore, must be sufficiently eliminated to guarantee safe usage of drinking water for potable and public purposes [1]. New technologies are in constant demand for the reduction and better complete removal of these harmful contaminants, in order to ensure the safe use of drinking water and significantly reduce the environment risk of water-borne diseases [2]. In recent years, membrane technologies have been favored over other traditional drinking water treatment technologies for removal of bacteria from drinking water effluents, such as disinfection, distillation, or media filtration, due to the advantages offered, such as high stability, high efficiency, low cost, low pollution, flexible equipment design, and small footprint [3,4,5,6].

Bacteria tend to form a cohesive biofilm on membrane surfaces, leading to an increase in the hydraulic resistance to permeation flow [7]. Therefore, several studies have developed improved biofouling control strategies for the more sustainable operation of membrane systems, it was demonstrated the potential of silver nanoparticles [7,8,9] and copper nanoparticles [10] as biocides, which can mitigate the membrane biofouling and restrain the flux decline effectively; the membrane systems subsequently showed no decline in hydraulic permeability in the whole membrane. Carbon nanotubes (CNTs) have also emerged as promising filtration and separation materials for water purification, owing to their strong antimicrobial activity, good corrosion stability, large active specific surface area, and excellent sorbent properties [11,12,13,14]. Multi-walled carbon nanotube (MWCNT) and single-walled carbon nanotube (SWCNT) membranes were used as self-supporting membranes, which were very effective for the complete removal of bacteria [15]. However, for the removal of bacteria in contaminated drinking water using carbon nanotube membranes, the first concern is focused on the mechanical properties of the free-standing membranes. The extremely high specific surface area and open porosity of the membranes offers a very high permeability and selectivity. However, it also exposes such membranes to higher mechanical stresses during long-term dynamic water filtration. As a result, the membrane could be easily compacted, deformed, or even destroyed during long-term filtration process, which, thus, significantly degraded the membrane structure and performance. CNTs constructed on microporous substrates with high mechanical property, such as ceramic membranes (spinel-based [16] and Al_2_O_3_ [17]), are considered to be very effective to solve the problem often encountered of the low mechanical strength of free-standing CNT membranes [18]. The excellent high-temperature-resistant property of ceramic membranes [19,20] make them very essential for the in situ controllable growth of CNTs by the chemical vapor deposition (CVD) method, at 500–800 °C, to form ceramic-CNT composite membranes. Due to excellent high temperature resistance and good corrosion resistance [21,22,23,24], mullite hollow fiber membranes can be considered as ideal substrates for in situ construction of CNTs.

The primary separation mechanism of the CNT membranes in the studies discussed above is based on size exclusion or sieving [25]. Such membranes often require high pressure for operation and are prone to pore plugging and performance deterioration upon filtration of environmental samples. Membrane fouling by attachment of bacteria on the membrane surface adds to the energy consumption and the complexity of the process design and operation. Furthermore, it significantly deteriorates membrane performance and reduces the lifetime of membranes and membrane modules. 

In this work, we demonstrate a CVD method to construct, in situ, highly efficient CNTs by direct growth of CNTs on a porous mullite ceramic substrate, and obtain a new form of mullite-CNT composite membrane with a porous membrane surface with a special randomly-oriented CNTs network structure featuring very high three-dimensional open porosity. The composite membrane exhibits unprecedentedly low fouling propensity and ultrahigh removal efficiency, by separation of *E. coli* and *S. aureus* from drinking water, during the 24 h filtration process.

## 2. Materials and Methods

### 2.1. Preparation of Mullite-CNT Composite Membrane

The mullite ceramic substrate, which was made from industrial solid-waste coal fly ash and bauxite mineral, was prepared in-house as described in our previous study [26]. The Ni catalysts coated on the mullite ceramic substrate were prepared by the one-step hydrothermal synthesis method according to the following procedure: 21 g Ni(NO_3_)_2_·6H_2_O were dissolved in 50 mL deionized water. Mullite ceramic substrate with Ni precursor was dried in air at room temperature, and then heated at a rate of 5 °C·min^−1^, from room temperature to 550 °C, and kept for 2 h; this resulted in the NiO-coated mullite ceramic substrate. The ratio of the sum of NiO (in weight) to mullite ceramic substrate was approximately 0.1:1 g; achieved by repeating the immersion–drying–calcination process.

Mullite-CNT composite membrane was prepared via a CVD method. Briefly, the CVD system consists of a horizontal quartz tube housed in a furnace. The NiO-coated mullite ceramic substrate was reduced at 500 °C for 1h with H_2_–N_2_ mixed gas at a flow rate of 40 mL·min^−1^. The reactor was heated up to 650 °C with pure methane gas at a 30 mL·min^−1^ flow rate, and maintained for about 2 h. The sample was naturally cooled to room temperature under the flow of N_2_ at a rate of 20 mL·min^−1^, and, accordingly, the mullite-CNT composite membrane was obtained.

### 2.2. Membrane Separation of Bacteria-Contaminated Drinking Water

Two common pollutants in drinking water are the model bacteria, *E. coli*, a rod-shaped bacterium having a typical length of 2000–5000 nm and width of 400–600 nm, and *S. aureus*, with a spherical size of ~1000 nm. They were selected to model Gram-negative and Gram-positive bacteria, respectively. Additionally, they are often used as indicators of pathogenic bacteria [27]. Colony counting methods were used to investigate the antimicrobic property of the membranes. Cultures of *E. coli* and *S. aureus* were shaken at 37 °C at 180 rpm for 24 h in a LB (lysogeny broth) medium until they reached the plateau phase. At this phase, the *E. coli* and *S. aureus* were rinsed twice using centrifugation, and then resuspended in a 0.9% (*w*/*w*) NaCl solution in preparation for the membrane separation experiments. 

A lab-scale membrane separation system was employed for the bacterial filtration experiments. The membrane was designed with an inside-out flow mode, and membrane filtration experiments were performed in dead-end filtration mode under 0.1 MPa trans-membrane pressure. The composite membrane was placed in a membrane holder and all the experimental materials were sterilized before use. An equal total number of *E. coli* cells (9.0 × 10^7^ bacteria per mL) and *S. aureus* cells (6.4 × 10^6^ bacteria per mL) were filtered through the mullite-CNT composite membrane. Before the filtration experiments were conducted, all the ceramic-CNT composite membranes were first pre-treated by tap water at 0.1 MPa for 1h. Membrane permeate samples were collected in an automated collector (BSZ-100, Shanghai Qingpu Huxi Co., Ltd., China). The permeate flow rate was recorded at 2 h intervals for 48 h. The feed and permeate were cultured on LB agar in solid media, and incubated at 37 °C for 24 h to determine the total bacterial count, which was determined by the plaque forming unit (PFU) method (US Environmental Protection Agency (EPA) Method 1601). All experiments were, at a minimum, duplicated at each dilution at room temperature. *E. coli* and *S. aureus* suspensions were filtered through the mullite-CNT composite membrane and fixed immediately, with 2.5% glutaraldehyde, after sieving onto the mullite-CNT composite membrane, and then dehydrated in preparation for scanning electron microscopy (SEM) analysis.

For all the membranes, the permeation flux (F, L·m^−2^·h^−1^) was calculated by Equation (1), where the volume of permeate (V, L), membrane area (A, m^2^), and time (t, h) were measured.

F = V/At(1)

The rate of dynamic retention of microorganisms of the composite membrane was defined as Equation (2):

R (%) = (1 − R_a_/R_b_) (2)

where R is the removal efficiency of bacteria, R_a_ is the number of the colonies on the plates after filtration, and R_b_ is the colonies number before filtration. 

### 2.3. Bacterial Inactivation Assay

The membranes were collected in the dark at a indicated time (shaking for 0, 2, 4, or 6 h) in saline solution at 37 °C. Assessment of the inactivated *E. coli* and *S. aureus* on the mullite-CNTs composite membranes was conducted by using the fluorescence-based Live/Dead^®^, BacLight TM Bacterial Viability Kit in combination with flow cytometry. Before flow cytometric analysis, the samples were filtered through a 20 μm filter to separate bacteria from bigger particles, and then cells were stained with the Live/Dead^®^, BacLight TM Bacterial Viability Kit (a mixture of SYTO^®^9 nucleic acid stain and PI (propidium iodide)). The inactivation percentage was determined by direct counting of PI-stained inactivated cells, divided by the total number of cells that were stained with PI plus SYTO-9. Flow cytometric measurement was performed on a flow cytometer (EPICS^®^ ALTRA™, Beckman Coulter Inc., Brea, CA, USA). 

## 3. Characterizations

SEM images were obtained using a field emission scanning electron microscopy (FESEM, S-4800, Hitachi Ltd., Tokyo, Japan). X-ray diffractometer (XRD, D8 advance, Bruker Corporation, Germany) patterns were measured at 2θ angle ranging from 10° to 80°. The crystalline structure of CNTs was measured using transmission electron microscope (TEM, JEM-2010; JEOL, Tokyo, Japan). Raman spectroscopy was performed in a wave number range of 100–3000 cm^−1^ using a Raman spectrometer (LabRam Aramis, Horiba Jobin Yvon, France) with an Ar-ion laser at 532 nm. Pore size distribution was measured by a pore size analyzer (PSDA-20, Nanjing Gaoqian Function Materials Co. Ltd., China), based on a liquid–liquid displacement bubble-point method. The concentration of metal ions in the filtrate was measured using ICP-MS (Inductively coupled plasma mass spectrometry, Nex ION 300D, PerkinElmer Inc., Shelton, CT, USA).

## 4. Results and Discussion

### 4.1. Construction of the CNTs Network on the Mullite Ceramic Substrate

**Figure 1:**
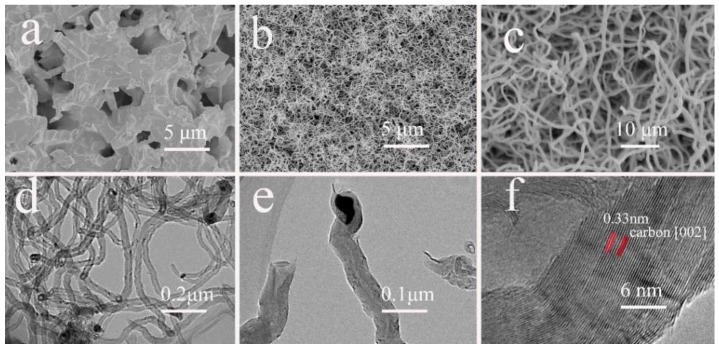
The characterization of the mullite-CNTs composite membrane. (**a**) scanning electron microscopy (SEM) image of original mullite ceramic substrate; (**b**) low magnification SEM image of mullite-CNTs composite membrane; (**c**) high magnification SEM image of mullite-CNTs composite membrane; (**d**) transmission electron microscopy (TEM) image of CNTs collected from the composite membrane; (**e**) CNTs with particle on tip; and (**f**) High resolution transmission electron microscopy (HRTEM) image of the CNTs.

As illustrated in Figure 1a, a hierarchical network was grown on the surface of the mullite ceramic substrate. The pristine mullite ceramic substrate had an uneven surface with randomly distributed spaces between the sintered mullite particles. The average pore size of the mullite ceramic substrate was found to be 1.02 µm, as reported in our previous study [26]. CNTs were successfully grown on the surface of the mullite ceramic substrate via the CVD method. The CNTs were intertwined with each other, forming a network-like hierarchical structure and were observed on the surface of the mullite ceramic substrate (Figure 1b,c). The special structure of the mullite-CNT composite membrane was expected to allow for high fluxes at low operating trans-membrane pressures due to the highly porous interlocking structure of the CNTs, which possibly could achieve a highly efficient separation of bacteria.

TEM results (Figure 1d) also clearly revealed that the resulting nanomaterials were hollow, being CNTs rather than solid carbon nanofibers, with an average diameter of 41 nm and lengths of up to several micrometers. Tip growth was the dominating mechanism of CNTs, and, as expected, catalyst particles could be detected at the tip of the nanotubes (Figure 1e), as described in the previous report [28]. The distance between two adjacent planes, d-spacing, was measured to be 0.33 nm, which was characteristic of the (002) plane of CNTs. The presence of metallic nickel in the CNTs layer revealed that the NiO phase was reduced during catalyst pretreatment. Nano-sized metallic nickel was quite active for the decomposition of methane [29].

**Figure 2:**
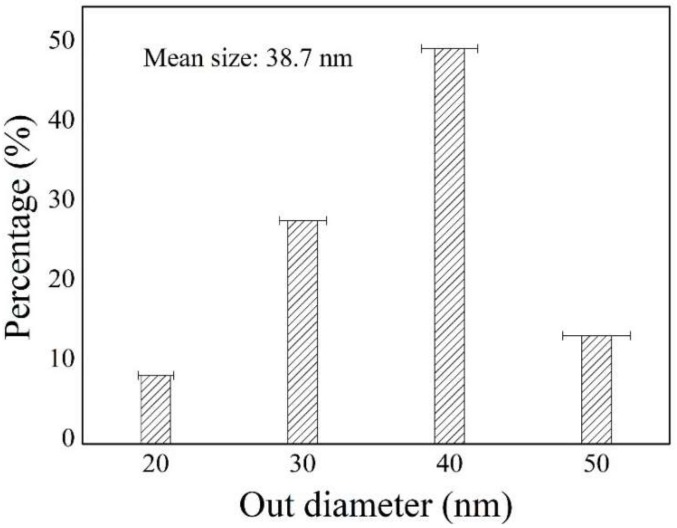
The diameter distribution of the CNTs in the mullite-CNT composite membrane.

Figure 2 presents the corresponding diameter distributions of the CNTs, measured from the SEM image in Figure 1c. The average diameter of the CNTs was ~38.7 nm, with a relatively narrow diameter distribution range of 20–50 nm.

### 4.2. XRD Analysis

**Figure 3:**
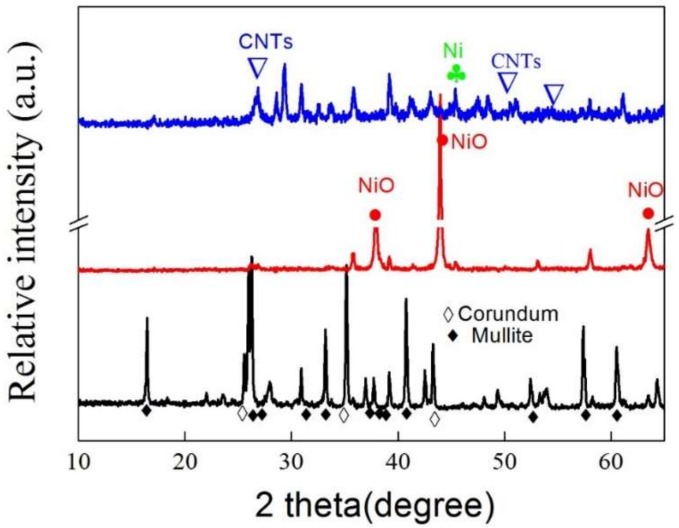
The X-ray diffractometer (XRD) patterns of the mullite ceramic substrate (black line), NiO catalysts on the mullite ceramic substrate (red line), and the mullite-CNT composite membrane (blue line).

The XRD patterns of the mullite ceramic substrate (black line), fresh NiO catalysts on the mullite ceramic substrate (red line), and the mullite-CNTs composite membrane (blue line) are given in Figure 3. It was indicated that the XRD pattern of the mullite ceramic substrate consisted of mullite (PDF#15-0776) and corundum (PDF#10-0173) characteristic peaks [19]. The decomposition of nickel nitrate, in the air at 550 °C for 2 h, to form the NiO species during the preparation of catalysts was verified by the red pattern, as shown in the Figure 3. From this, it was concluded that only NiO phase was present in the fresh catalysts, and that the mullite-CNT composite membrane displayed phases due to both metallic nickel and the graphitic carbon (blue line).

### 4.3. Raman Spectroscopy

**Figure 4:**
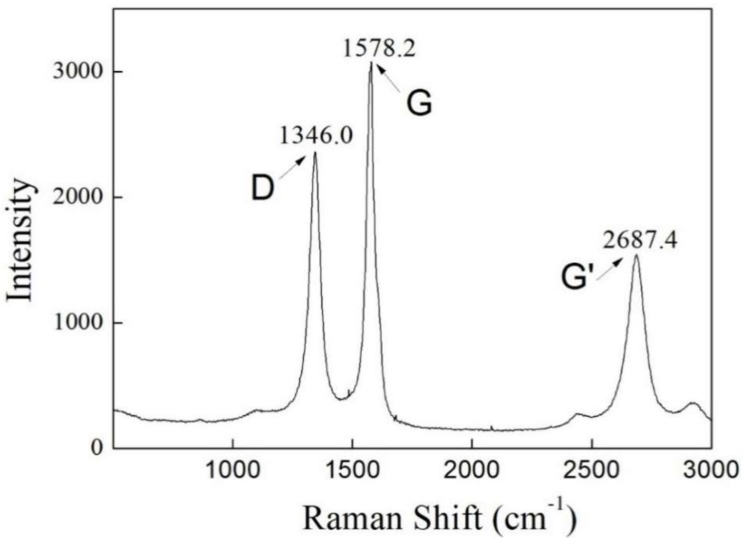
Raman spectrum of the as-fabricated mullite-CNT composite membrane.

To accurately identify the nature of the formed CNTs, we identified the CNTs (MWCNTs) by Raman spectroscopy. Figure 4 shows the Raman spectrum of the as-grown CNTs on the mullite substrate. The G-line corresponds to the E_2g_ mode (i.e., the stretching mode of the C–C bond in the graphite plane), demonstrating the presence of crystalline graphitic carbon with a sp^2^ orbital structure. The D-line, centered around 1346 cm^−1^, originates from disorder in the sp^2^-hybridised carbon and can be ascribed to the presence of lattice defects in the graphite sheets that construct CNTs. Since the D band is an intrinsic feature of multi-walled carbon nanotubes (MWCNTs), the CNTs formed in this study were determined to be multi-walled CNTs (MWCNTs).

### 4.4. Pore Structure

**Figure 5:**
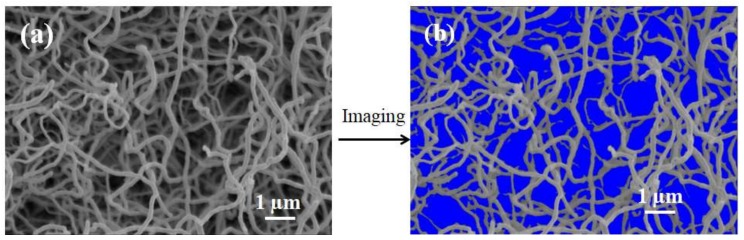
Surface SEM images of the membrane by Image J software (Version:3.4, National Institutes of Health Bethesda, MD, USA) (**a**,**b**); the blue area in (**b**) denotes surface porosity.

The surface porosity of the membrane was quantified using the tool Image J, as shown in Figure 5, and showed a high value of 56.7%.

**Figure 6:**
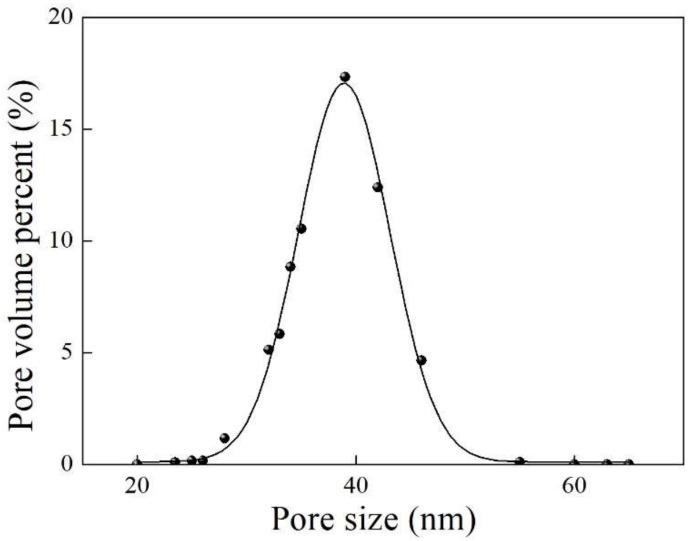
Pore size distribution of the mullite-CNT composite membrane.

The pore size distribution of the mullite-CNT composite membrane is shown in Figure 6. The average pore size of the mullite substrate was 1.02 μm [26]. After the CVD process, CNTs were uniformly grown on the surface and inside the macro-voids long-channels of the finger-like layer of the composite membrane, resulting in a lower average pore size, down to ~40 nm, as shown in Figure 6.

### 4.5. Highly Stable Flux for Bacterial Removal

**Figure 7:**
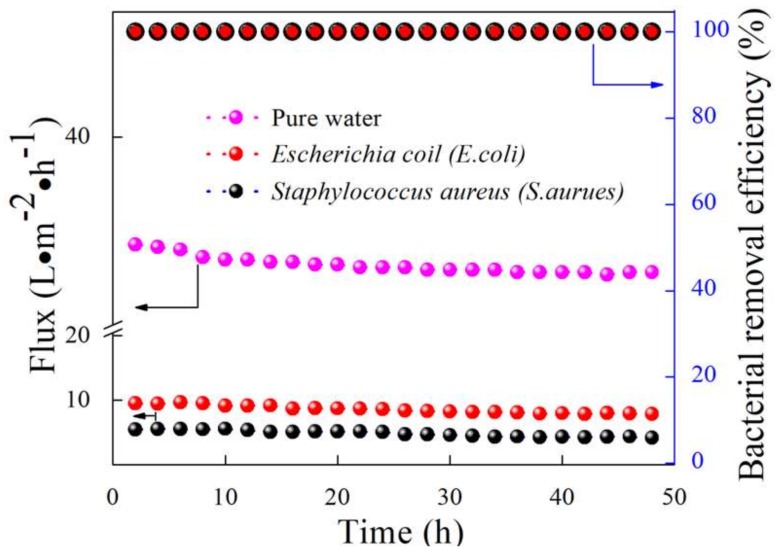
Flux and bacterial removal efficiency, as a function of the operation duration, of the mullite-CNTs composite membrane.

As presented in Figure 7, the pure water flux was 38.7 L·m^−2^·h^−1^, which was higher than the bacteria–water flux due to the absence of bacteria. The mullite-CNT composite membrane was demonstrated to effectively remove bacteria from drinking water, with a highly stable long-term flux, for the 24 h filtration process, which was driven b­y a trans-membrane pressure of 0.1 MPa. The initial permeate fluxes of the mullite-CNTs composite membrane were 9.5 L·m^−2^·h^−1^ and 5.4 L·m^−2^·h^−1^ for *E. coli* and *S. aureus*, respectively, at 0.1 MPa. Subsequently it was characterized by a very mild decrease in fluxes, of which values were 7.9 L·m^−2^·h^−1^ and 4.4 L·m^−2^·h^−1^ of *E. coli* and *S. aureus*, respectively, at 48 h of operation. The fluxes of *E. coli* and *S. aureus* were decreased by 17.3% and ~19.2%, respectively, during the filtration period. During the long filtration experiment, the fluxes of the composite membrane decreased slightly and showed excellent performance, with outstanding efficiency in the removal of bacteria, as illustrated in Figure 7; *E. coli* and *S. aureus* have been eliminated totally based on the PFC method at 2 h, 24 h, and 48 h.

**Figure 8:**
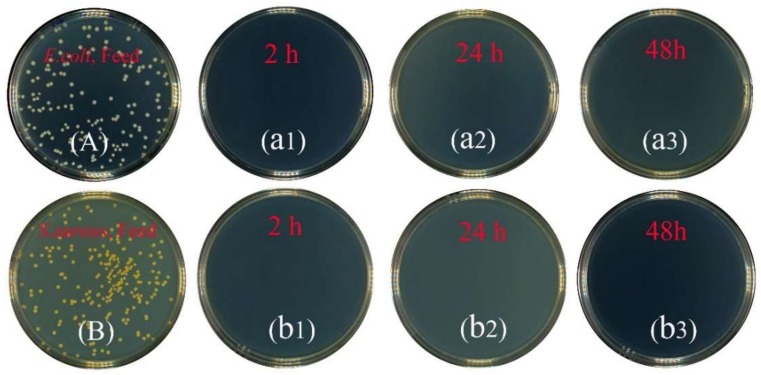
Digital images of plaque forming unit (PFU) results. The feed containing *Escherichia coli* (*E. coli*, (**A**)) and *Staphylococcus aureus* (*S. aureus*, (**B**)); the filtrate after different treatment times of 2 h, 24 h, and 48 h for *E. coli* (**a1**–**a3**) and *S. aureus* (**b1**–**b3**).

**Figure 9:**
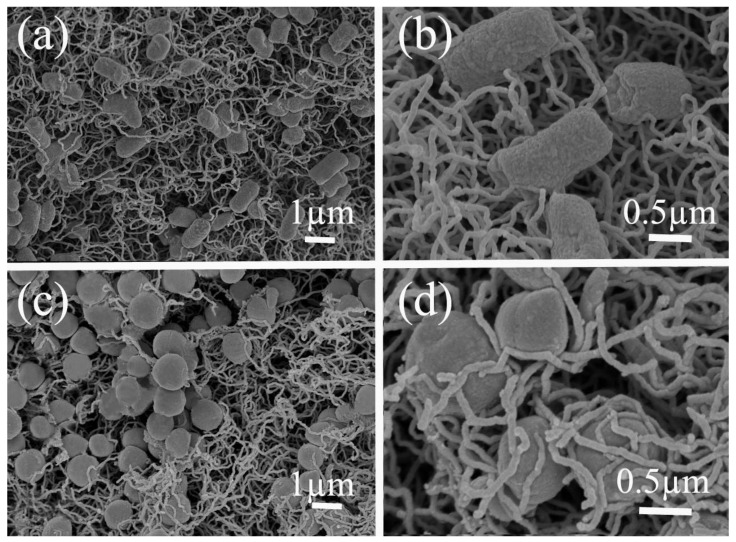
SEM images of *E. coli* (**a**,**b**) and *S. aureus* (**c**,**d**) on the mullite–CNT composite membrane after the filtration. High magnification view of the composite membrane with the captured *E. coli* (**b**) and *S. aureus* (**d**).

The results showed that the removal of bacteria from the 10^7^ per mL initial concentration was complete, without any bacteria detected by the PFU method at the filter outlet. High levels (100%) of removal of bacteria, *E. coli* and *S. aureus*, were shown in Figure 8 throughout the filtration experiment. Additionally, it showed that the mullite-CNT composite membrane could be used successfully to obtain bacteria-free water at a long operation time. The results were due to the formation of a “network-like” hierarchical structure [30] by the CNTs on the mullite ceramic substrate. The bacteria were only trapped on the CNTs network surface via surface filtration, without pore plugging, endowing the mullite-CNTs membranes with unprecedentedly low fouling propensity in order to keep high flux with operation time. To confirm this and to check the separation mechanism of the mullite-CNTs membranes for the two model bacteria, SEM images of *E. coli* (Figure 9a,b) and *S. aureus* (Figure 9c,d) on the mullite-CNT composite membrane after the filtration are discussed below.

The crosslinked CNTs network formed unique shape and pore systems, confirmed by the SEM pictures as shown in Figure 9a,c. Figure 9a,c also demonstrated that the mullite-CNT composite membrane was porous. The pore size of the mullite-CNTs composite membrane was very small compared to the size of bacteria, so bacteria cannot permeate through the membrane; only water molecules were allowed to permeate through the membrane pores. Higher porosity indicated that a higher number of pores and channels were available for water transportation. SEM images also indicated that the *E. coli* and *S. aureus* cells were completely retained by the CNTs layer, by a sieving mechanism, due to size exclusion. The CNTs membranes for bacteria removal based on size exclusion or sieving, often required high pressure for operation, which were prone to pore plugging and performance deterioration upon filtration of environmental samples in the studies discussed before. A major advantage of using the mullite-CNT composite membrane, over conventional membranes, lies in the high flux with excellent removal efficiency over a long period of time. 

At higher magnification, Figure 9b,d, it was found that *E. coli* and *S. aureus* cells were captured by the tangled CNTs networks. The bacteria were removed along with a surface-filtration, not via in-depth pore blocking, which would lead to a rapid decrease flux depth-filtration mechanism, that is, captured by the CNTs on the surface of the mullite-CNT composite membrane. Accordingly, the high flux was retained throughout the filtration time due to the formation of the porous membrane surface with a special randomly-oriented CNTs network structure, featuring very high three-dimensional open porosity, allowing water to rapidly transport in any direction. The high flux was also predicted by the “flow enhancement” of the slip flow due to the small diameter of the CNTs [31]. The mullite-CNTs composite membrane was more effective in capturing the bacteria on the surface, but not in the pore channels of the composite membrane. This suggests that the filtration step may have little effect on the water permeation flux without blocking the pores, thus overcoming the inherent limitation of the trade-off effect between high removal efficiency and high flux.

**Figure 10:**
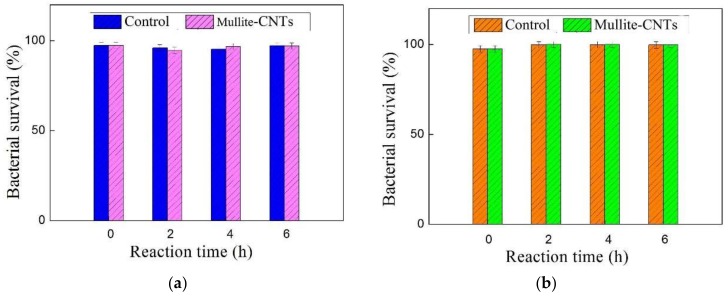
*E. coli* (**a**) and *S. aureus* (**b**) survival after exposure to mullite ceramic substrate (control) and mullite-CNT composite membrane.

A fluorescence-based viability kit (Live/Dead), as shown in Figure 10, was used to determine the percentage of *E. coli* (a) and *S. aureus* activated on the mullite-CNT composite membrane and the mullite ceramic substrate, used as control samples for the comparison. The two bacteria, *E. coli* and *S. aureus,* demonstrated greater resilience to exposure to the CNTs membrane in this study, which was not consistent with the results reported dealing with CNTs toxicity tests on bacteria physiology [32,33]. The two hour exposure time led to inactivation of only less than 1% for both the two bacteria; the inactivation of bacteria on the CNTs membrane did not increase significantly over the longer incubation time of 6 h exposure to the mullite-CNT composite membrane. Studies have shown that MWCNTs were less effective than SWCNTs in cell inactivation, the top layer in this study was MWCNTs, so the effect may be not as evident as reported by the literature [32,34]. It has been hypothesized that direct contact with the CNTs was necessary to attain inactivation. The carbon nanotubes were fixed on the surface of the mullite ceramic substrate, which provided a small contact area with the bacteria, and the contact time between the membrane surface and the filtrate was relatively short. Due to the short contact time and small contact area, between the membrane surface and the bacteria, the MWCNTs layer and the metallic nickel showed less antimicrobial activity towards *E. coli* and *S. aureus*, and the influence of the prepared mullite-CNT composite membrane on the inactivation of the trapped bacteria was negligible.

Release of CNTs into the environment is a major concern. To assess any possible environmental risk of the developed ceramic-CNT membrane in water treatment due to the existence of some metal ions, such as Al and Ni, in the membrane starting materials, a gas-cleaning run was conducted to ensure that no CNTs were unbound from pore channels under the shear stress of water. The selected permeated water samples were examined by SEM and no CNTs were found. Additionally, the concentration of these metal ions in the filtrate was measured using ICP-MS (inductively coupled plasma mass spectrometry, Nex ION 300D, PerkinElmer Inc., USA). The results shown in Table 1 clearly show very low levels of these metal ions, consistently meeting the standard of drinking water criterion issued by the World Health Organization (Guidelines for Drinking-Water Quality Fourth Edition, WHO, 2011).

Therefore, the release of CNTs from the membrane might not be of a concern for the impact on the environment and ecosystems. The mullite-CNT composite membrane was able to stand a pressure difference of 0.1 MPa, suggesting that a pressure difference of 0.1 MPa does not weaken the integrity of the membrane. Thus, it was supposed that the strong adhesion force between the CNTs layer and the porous mullite ceramic substrate was important for long-term operation in water treatment application. 

## 5. Conclusions

In this work, a porous mullite-CNTs composite membrane, where the CNTs were in situ immobilized by chemical vapor deposition on a hierarchically-structured mullite ceramic substrate. The composite membrane exhibited unprecedentedly low fouling propensity and ultrahigh removal efficiency (100%), by separation of two model bacteria (*E. coli* and *S. aureus*) from drinking water, with highly stable long-term flux for the 24 h filtration process, which was driven by a trans-membrane pressure of 0.1 MPa. The fluxes of separation of *E. coli* and *S. aureus* were decreased by only 17.3% and ~19.2% during the filtration period. The enhancement was due to the formation of a porous membrane surface with a special randomly-oriented CNTs network structure, featuring very high three-dimensional open porosity, allowing water to rapidly transport across the membranes. The bacteria were only trapped on CNTs network surface via surface filtration, without pore plugging, endowing the mullite-CNT membranes with unprecedentedly low fouling propensity in order to keep high flux with long operation time. 

## Figures and Tables

**Table 1:** Concentration of selected metal ions in the filtrate.

Concentration (mg·L^−1^)	Al	Ni
CNTs membrane	0.07	nd
Drinking water criterion (WHO)	0.2	0.07

nd—not detected.
